# Counselling and knowledge on iron and folic acid supplementation (IFAS) among pregnant women in Kiambu County, Kenya: a cross-sectional study

**DOI:** 10.12688/aasopenres.12891.3

**Published:** 2019-05-13

**Authors:** Mary Kamau, Samuel Kimani, Waithira Mirie

**Affiliations:** 1 School of Nursing Sciences, University of Nairobi, Nairobi, Kenya

**Keywords:** Pregnancy, Iron and folic acid supplementation, Knowledge, Counselling information, Anaemia

## Abstract

**Background: **The increased demand for iron and folic acid during pregnancy is not met through diet due to insufficiency or reduced bioavailability of nutrients among women from low income countries. Thus, iron and folic acid supplementation (IFAS) is a promising interventional strategy for control of anaemia during pregnancy. Kenya adopted the global IFAS intervention with a target of 80% coverage by 2017, however, the compliance remains low. Increasing awareness, counselling, communication and community education on IFAS have improved compliance among pregnant women. Thus, we aimed to determine: IFAS knowledge, availability, practices, and content of IFAS counselling among pregnant women attending health facilities in Kiambu County, Kenya.

**Methods: **A cross-sectional study involving 364 pregnant women aged 15-49 years. A two stage cluster sampling, including one sub-county and five public health facilities were used. A pre-tested, structured questionnaire consisting of socio-demographic data, maternal knowledge and counselling on IFAS was used. An observation checklist was used to observe practices and content of antenatal counselling session in each facility. Data was analysed using STATA in which descriptive and inferential statistics were computed.

**Results: **Of 364 respondents, less than half (40.9%) scored high on knowledge on IFAS. Women who were counselled on duration of IFAS intake, side effects, and their mitigation were more likely (p <0.005) to have high IFAS knowledge. Although all the health facilities had varied IFAS posters displayed, none had key IFAS counselling documents.

**Conclusion: **Less than half of the pregnant women had high IFAS knowledge, IFAS documents were scarce in health facilities, IFAS counselling information in different health facilities was limited and varied, and content of counselling was associated with levels of knowledge on IFAS. This underscores the need to strengthen focused and targeted IFAS counselling for pregnant women and standardization of counselling messages to improve compliance and pregnancy outcomes.

## Introduction

Nutritional status during conception and pregnancy is a predictor of maternal and infant outcomes ^1^. Pregnancy increases metabolic activity including demand for macro and micro nutrients, particularly iron and folic acid. The levels of body stores for most critical nutrients, particularly iron and folate, are usually suboptimal by the time of conception among most women in developing countries, thus their requirement is greater resulting in a need for supplementation. This implies that a slight decrease in haemoglobin levels in pregnant women can cause anaemia which can result in severe and often fatal consequences especially if not addressed early ^2^. The consequences include increased risk of mortality, morbidity, postpartum haemorrhage, and poor birth outcomes, including foetal growth retardation, preterm births and low birthweight ^3– 5^.

Anaemia in pregnancy is a leading cause of global burden of disease ^2^ with iron deficiency anaemia being responsible for more than half of the cases. The global prevalence of anaemia in pregnancy ranges from 41.8–43.8% with the greatest (61.3%) burden being found in Africa then South East Asia at 52.5% ^6, 7^. Africa has the highest prevalence of anaemia among both pregnant and non-pregnant women while Asia has the largest absolute number of women with anaemia, forming 38% of the global total ^8^. In Kenya, anaemia in pregnancy remains a public health problem, with the prevalence being persistently high, currently at 55.1% ^2, 7, 9^ resulting in estimated 10% maternal deaths and 20% perinatal deaths ^10^.

Iron and folic acid supplementation (IFAS) is one of the most affordable and effective global intervention strategy for control of anaemia in pregnancy with resultant benefits of reduced maternal-child morbidity and mortality ^10– 12^. This is necessitated by the fact that the high body’s nutrient demand in pregnancy is not met by regular diet because of insufficient amounts and/or low bioavailability in diets ^5^. Following the WHO guidelines ^13^, Kenya adopted IFAS programme targeting to achieve 80% coverage by 2017 ^10^. Indeed, the IFAS tablets are currently routinely provided through all public health facilities during antenatal care, free of charge for daily use throughout pregnancy. However, the government’s effort to provide IFAS for free notwithstanding, compliance remains low over the years. Reports show that only about 8% pregnant women take IFAS for more than 90 days ^14– 17^. Studies indicate that poor compliance hinders IFAS success with subsequent poor maternal-child outcomes ^12, 18, 19^.

Many factors are substantially associated with the non-use of IFAS including: ineffective management, limited funding, stock-outs, maternal age, maternal literacy, spouse literacy, wealth index, frequency of antenatal care (ANC), comprehensive knowledge of anaemia, and quality of counselling on IFAS during pregnancy ^20, 21^, among others. At the heart of these factors lies lack of demand from health sectors and beneficiaries ^22^. Studies have shown that increasing awareness on IFAS, appropriate counselling, focused communication and community education among pregnant women improve IFAS compliance ^23– 26^. Information and training on importance of supplementation during pregnancy is associated with better IFAS utilization ^20, 27^ in terms of longer duration ^28^ and increased compliance ^29^, eventually leading to more effective supplementation ^30^. There is need to develop appropriate counselling strategies to address this poor compliance.

Counselling is one of the strategies for Social Behaviour Change and Communication (SBCC). Social behaviour change and communication is an evidence based communication style aimed at influencing observable, measurable actions so as to improve the health of individuals and communities ^31, 32^. The use of Behaviour Change and Communication (BCC) strategies including counselling to improve health outcomes over the years cannot be overemphasized ^33^. The BCC strategies are particularly effective when designed and implemented locally: tailored to the local context realities and locally accepted, owned and driven ^34^. In Kenya, various strategies have been integrated to different programmes to develop and disseminate evidence based communication campaigns to influence behaviour change on diverse health issues including communicable diseases control especially HIV/AIDS, child welfare services, non-communicable diseases control, health promotion, various maternal issues and nutrition and supplementation programmes, among many others. For example, Malaria Trac 2014 showed a strong correlation between exposure to Interpersonal Communication and net use behaviour ^35^.

Counselling applies interpersonal communication at different levels to influence individual and collective behaviours that promote health. These can occur by producing changes in a wide range of behaviours including knowledge, attitudes and social norms, practices and many others ^36, 37^. For the behaviour change to occur and have these changes fully internalized, clients need to move from the level of information to motivation and then progress to experimenting with behaviour change ^38^. Thus counselling provides an excellent opportunity for interacting with the client through interpersonal communication to achieve behaviour change.

Various factors affect delivery and uptake of counselling messages, related to either the service-provider, health facility or client. In order to deliver quality counselling for behaviour change, basic counselling skills, communication skills and therapeutic approach are key ^39^. Service-provider factors include: knowledge, skilfulness, ability to translate conceptual ideas into the local context and interpersonal communication skills, among other counselling skills ^40^. Health facility factors include infrastructure and equipment, workload, availability of IEC materials and job aids such as counselling cards, flyers, and posters. Client related factors include their perception, accessibility and affordability of the services ^41^. To ensure that quality counselling is provided and sustained, ongoing training and supervision of service-providers as well as routine assessment of practices and content of counselling messages given is essential.

Previous Kenyan studies have shown limited knowledge about anaemia or the importance of taking IFAS ^2, 29, 42, 43^. Information on the quality of counselling in association to maternal knowledge on IFAS in Kenya is scarce. Thus, the aim of this study was to: (1) determine maternal knowledge on iron and folic acid supplementation (2) determine availability of IFAS counselling documents in health facilities (3) assess practices and content of IFAS counselling information provided to antenatal women in health facilities and (4) determine the association between content of counselling information and level of maternal knowledge on IFAS.

## Methods

This was a cross-sectional study conducted between June and October 2016 involving 364 pregnant women, from Kiambu County, Kenya. Using two stage cluster sampling, one Sub-County (Lari) and five of its major public health facilities (Lari, Githirioini, Kagwe, Kagaa, and Kinale) were selected for the study. The Sub-County was selected on the basis of having existing functional community units (with active community health volunteers in health care activities implying completeness in health care provision at level one of health care service delivery). The study population consisted of all pregnant women who attended antenatal care in the selected health facilities who were: aged 15–49 years, below 33 weeks in their pregnancy gestation, not suffering from any chronic illness and who provided informed consent to participate in the study.

The minimum sample size was 285, as determined using Fisher’s formula (1999) [n = Z2pq/e ^2^] for a cross-sectional study, using the prevalence of IFAS compliance obtained from Thika hospital, Kiambu County among women attending ANC clinic, where n is the minimum sample score (z = 1.96), p is the presumed prevalence of IFAS compliance (24.5%), q (1-p) is the proportion of non-compliance and e is the margin of error (e = 0.05). A non-response rate of 30% was factored in and added to the minimum sample to make a total sample of 370. The study achieved a sample size of 364 which is 98.5% response rate. A structured interviewer-administered questionnaire ( Supplementary File 1) consisting of 24 closed ended questions including; 11 on socio-demographic data, 9 on maternal knowledge and 4 on counselling content on IFAS, was developed, pre-tested and used for data collection in this study. In addition, an observation checklist ( Data set 2) was used to determine availability of IFAS counselling documents in each health facility as well as counselling practices and content of information covered during an antenatal counselling session. To address any potential bias in data collection, training of four research assistants on research ethics and protocol and quality data collection was done at Kiambu level 5 hospital where the research questionnaires were pretested. To ensure reliability of the questionnaire, a test re-test method was adopted in pre-testing, whereby a repeat pre-test was conducted after two weeks, and Cohen’s kappa statistic was used to measure the level of agreement of the results from the two pre-tests. The questions which were tested and re-tested included: on socio-demographic data: age, education level, occupation, income, gestation, parity and gravidity; on maternal knowledge: benefits of IFAS, frequency of taking IFAS, duration of taking IFAS, possible side effects of IFAS, how to manage the side effects, food sources that increase blood levels, consequences of not getting enough iron/folate, and signs and symptoms of anaemia. All the questions repeated had a kappa value of above 0.7 after comparison thus the questionnaire was considered reliable, hence all the questions were retained. To ensure validity of the questionnaire, it was shared and discussed with experts from the Ministry of Health, division of nutrition, and the study supervisors. The feedback obtained from these experts and pre-testing results was used to refine the tool and improve its quality to ensure the questions were able to test what was intended.

The trained research assistants administered questionnaires to all pregnant women who met the inclusion criteria and consented to participate in the study at the health facilities selected for the study. Filling of the observation checklist was done by the researcher. One counselling session was observed in each health facility. To control for Hawthorne effect, discretion was applied whereby the investigator did not out-rightly inform the nurse that she was being observed but rather objectively recorded details of the counselling session on a notebook then immediately after filled the checklist objectively by stepping out of the antenatal room. The variables of this study were as follows: outcome was maternal knowledge on IFAS; the predictors of maternal IFAS knowledge were: socio-demographic characteristics, practices and content of counselling on IFAS; and availability of counselling documents was an effect modifier.

In regard to analyses, maternal knowledge was computed by summing up all relevant 40 Likert scale items (5 on benefits, 7 on possible side-effects of IFAS, 6 on managing side effects, 6 on effects of iron/folate deficiency, 7 on features of anaemia, 7 on dietary sources that increase blood levels, one item on frequency and one item on duration of IFAS). A correct answer for each item was scored as “1” and “0 for the incorrect response. All the scores for each respondent were summed up to determine the respondents’ level of knowledge. The frequencies of the observation checklist items were entered and data summarized into percentages. A cross tabulation of the data was then carried out against socio-demographic characteristics and content of counselling information offered at health facilities to determine their relationship.

Data from questionnaires was entered into SPSS version 20.0 and exported to STATA version 13.0 then the descriptive and inferential statistics were computed ( Dataset 1 ^38^). Eight of the questionnaires had missing data and were not included in the analysis. Univariate and multivariate binary logistic regression analysis was performed in order to identify the association between maternal IFAS knowledge and content of counselling information. All variables with P<0.05 during the univariate analysis were fitted in the multivariate analysis to identify variables independently associated with maternal IFAS knowledge. A 95% CI with respective odd ratios was used to assess the statistical significance of association among variables. The significance level was set at p <0.05. Descriptive statistics and binomial exact 95% confidence interval (95%CI) of proportions were used for reporting.

Ethical approval of the study protocol was sought and granted by Kenyatta National hospital/University of Nairobi Ethics and Research Committee (KNH-ERC/A/90 protocol number – P706/11/2015). Research permit was obtained from the National Commission for Science, Technology and Innovation (NACOSTI/P/18/81499/2231). Authority to conduct the study was obtained from Kiambu County, Lari Sub-county authorities and health facilities involved. All study participants provided verbal and written informed consent. The minors (below 18 years of age) who agreed to participate in the study provided verbal and signed assent and their parents provided an informed consent on their behalf. The STROBE cross sectional reporting guidelines were used for reporting ( Supplementary File 2) ^40^.

## Results

### Socio-demographic characteristics and knowledge on IFAS among respondents

Of the 364 respondents, 67.7% were aged 19–29 years, with the mean age of 25 years. Whereas 37.4% of the respondents had attained upper primary education, only 40.4% had completed secondary education and beyond. A majority (84.1%) of the respondents were married, and had 1–2 children (77.6%). Regarding economic activity, the respondents reported being housewives (27.4%), self-employed (25.1%), casual labourers (22.4%), and on formal employment (2.8%), respectively. Furthermore, only 7.8% of them reported earning more than 100 USD per month.

The distribution of the knowledge scores yielded a mean of 6.24 (SD=3.64) and a median of 6.00. Since the mean was slightly different from the median in this distribution, it shows that the distribution of the knowledge scores was not exactly normal. A further test of normality was done to determine the distribution of maternal knowledge scores using Shapiro-Wilk W test. The results of the test were statistically significant (W=0.961, p<0.001), which showed that the distribution of maternal knowledge scores was not exactly normal (rejecting the null hypothesis that distribution of maternal knowledge scores was normal). Thus, the median was used as the cut-off between high and low IFAS knowledge as in the study by Arega Sadore and colleagues ^44^. Those who scored above the median value were classified as having high IFAS knowledge, while those who scored below the median value were classified as having low IFAS knowledge ^45^.

The level of knowledge on IFAS among respondents was varied, with only 40.9% scoring an overall high. About two-thirds (67.3%) of them had heard of IFAS. Among those who scored high for IFAS knowledge, the highest percentage was among those on formal employment (70%) and aged above 40 years (66.7%) ( Table 1).

**Table 1.** Socio-demographic characteristics and level of knowledge on iron and folic acid supplementation (IFAS) among respondents.

Socio-demographics Characteristics		Level of IFAS knowledge	
	Overall n (%)	Low n (%)	High n (%)
**Age in years (n=356):** ≤ 18 19 – 29 30 – 39 40 – 49	28 (7.9) 241 (67.7) 84 (23.6) 3 (0.84)	23 (82.1) 151 (62.7) 38 (45.2) 1 (33.3)	5 (17.9) 90 (37.3) 46 (54.8) 2 (66.7)
**Marital Status(n=364):** Married Single Widow/Separated/Divorced	306 (84.1) 54 (14.8) 4 (1.1)	173 (56.5) 39 (72.2) 3 (75.0)	133 (43.5) 15 (27.8) 1 (25.0)
**Education Level (n=361):** Lower primary Upper primary Secondary incomplete Secondary complete Tertiary	8 (2.2) 135 (37.4) 72 (19.9) 125 (34.6) 21 (5.8)	3 (37.5) 86 (63.7) 44 (61.1) 67 (53.6) 12 (57.1)	5 (62.5) 49 (36.3) 28 (38.9) 58 (46.4) 9 (42.9)
**Occupation (n=362):** Formal employment Self-employed Casual labourer Housewife Students Unemployed Farming	10 (2.8) 91 (25.1) 81 (22.4) 99 (27.4) 11 (3.0) 55 (15.2) 15 (4.1)	3 (30.0) 51 (56.0) 54 (66.7) 53 (53.5) 9 (81.8) 36 (65.5) 7 (46.7)	7 (70.0) 40 (44.0) 27 (33.3) 46 (46.5) 2 (18.2) 19 (34.6) 8 (53.3)
**Number of children (n=237):** 1 – 2 3 – 4 5+	184 (77.6) 46 (19.4) 7 (3.0)	101 (54.9) 23 (50.0) 3 (42.9)	83 (45.1) 23 (50.0) 4 (57.1)
**Average income (n=351):** ≤ 10,000 Above 10,000	325 (92.6) 26 (7.4)	199 (61.2) 10 (38.5)	126 (38.8) 16 (61.5)
**Overall maternal IFAS knowledge**	364 (100)	215 (59.1)	149(40.9)

### Availability of IFAS counselling documents at the health facilities

**Figure 1.**
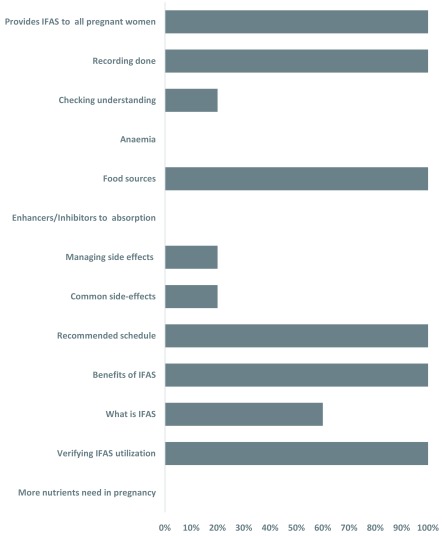
Observed counselling practices and content of counselling information provided. These are the counselling practices and content of counselling information provided to pregnant women by health care providers, as observed during an antenatal counselling session at each health facility, by the researcher.

All health facilities displayed diverse posters with information about IFAS at different service delivery points. Among the five facilities, only two displayed the IFAS national policy guidelines, while none had IFAS information, communication and education (IEC) materials, including health workers’ counselling guides, mothers’ calendars or brochures/leaflets on IFAS, among others ( Figure 1 and Dataset 2 ^38^).

### Observed counselling practices and content of information on IFAS at health facilities

A provider-client interaction observed during an antenatal counselling session in each health facility revealed that health care providers provided IFAS services to all pregnant women without discrimination, regardless of their haemoglobin levels. Verification of IFAS utilization and recording of IFAS services was done in all the health facilities. However, evaluation on the comprehension of the counselling content provided to clients only took place in one facility. Furthermore, counselling women on side-effects of IFAS and their management was only done in one facility. Despite the universal provision of IFAS to all antenatal women, they were not given information on the causes, features or consequences of anaemia in any of the health facilities. Moreover, the counselling did not include; the enhancers/inhibitors of iron/folate absorption, and the fact that there is increased nutritional requirement during pregnancy, in any of the health facilities ( Figure 1 and Dataset 2 ^38^).

### Relationship between IFAS counselling content and maternal knowledge on IFAS

**Figure 2.**
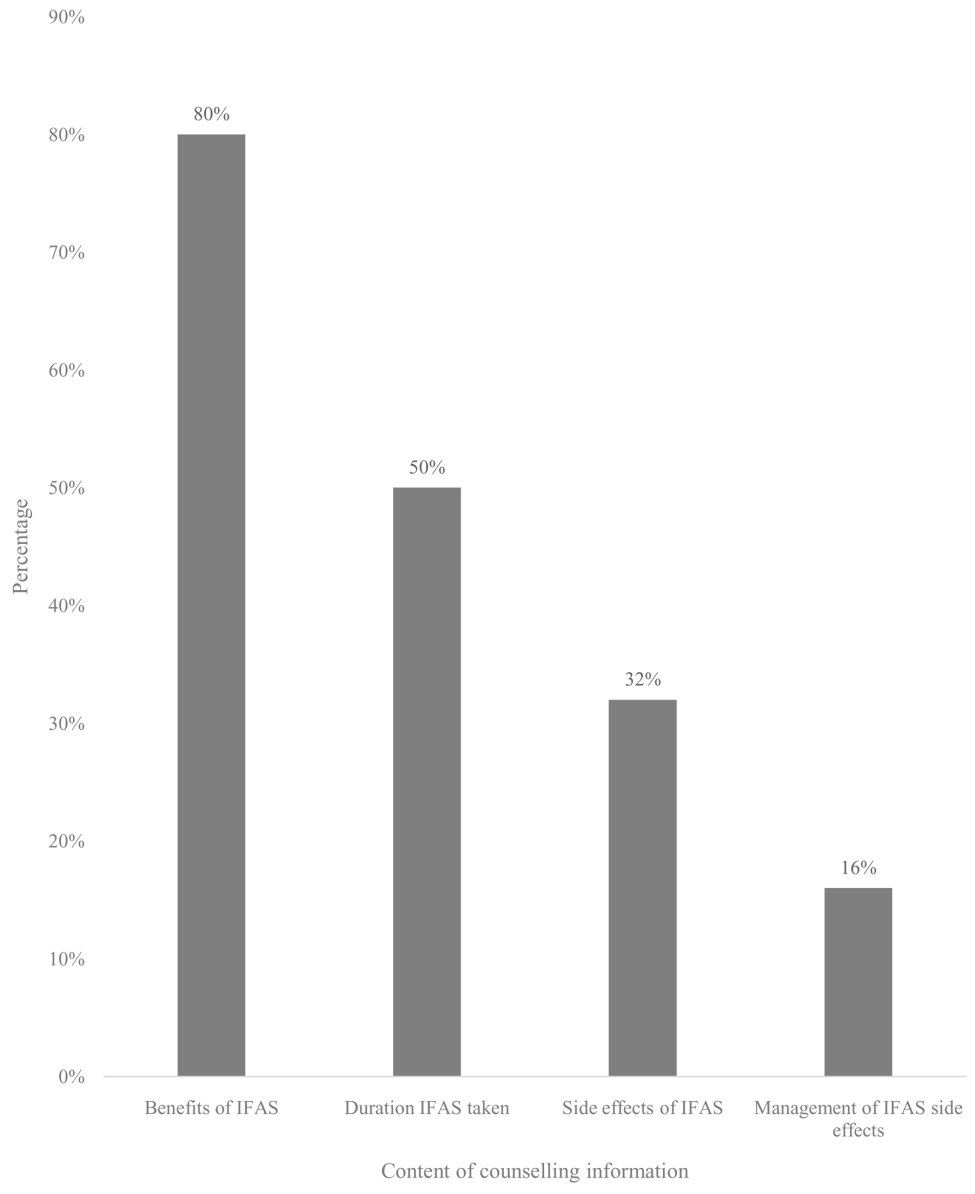
Content of counselling information provided on iron and folic acid supplementation (IFAS). This refers to the proportion of pregnant women counselled on various aspects of the content of IFAS counselling information, according to the women’s interviews.

**Figure 3.**
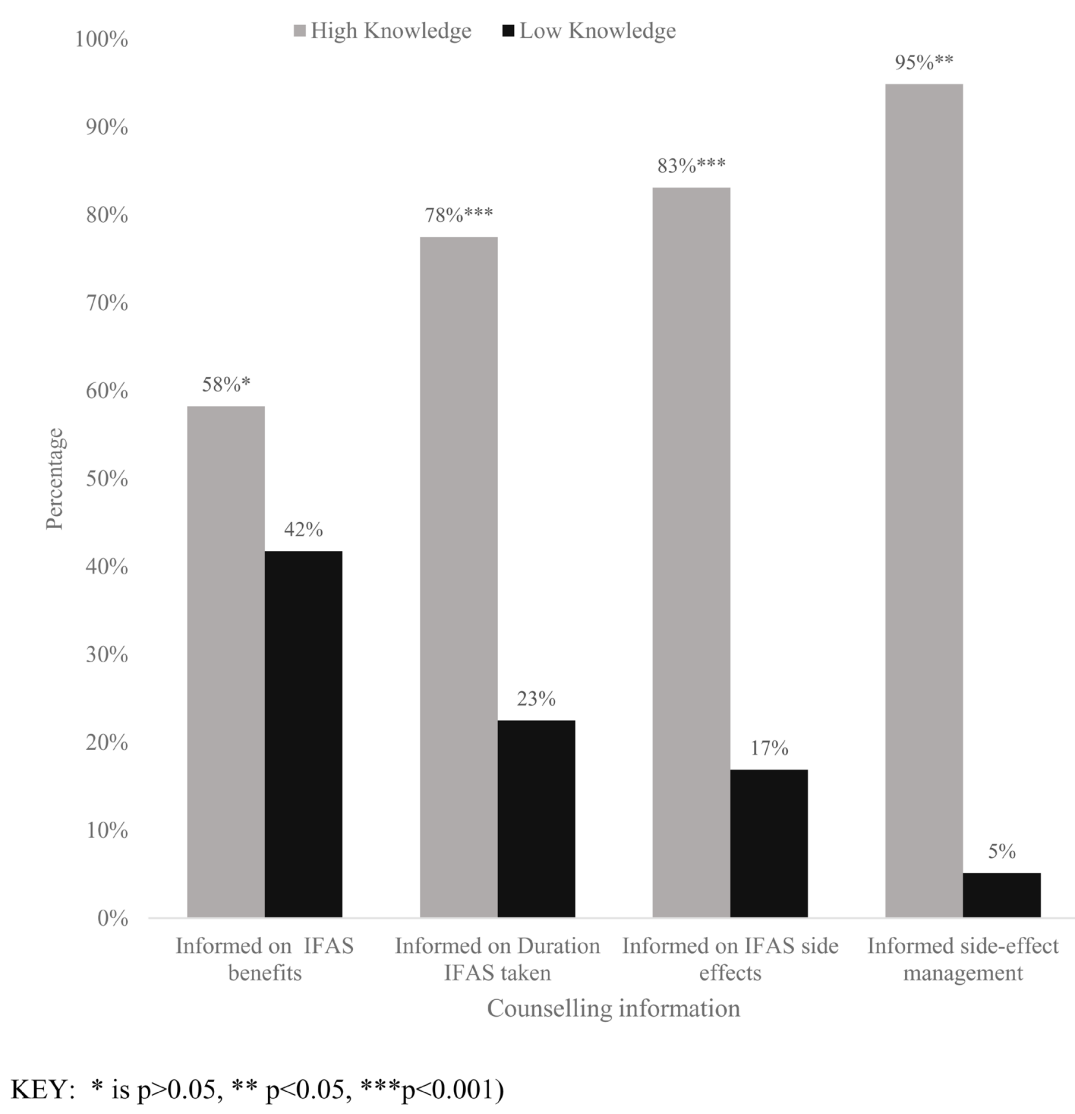
Relationship between counselling information and level of iron and folic acid supplementation (IFAS) knowledge. The proportion of pregnant women provided with IFAS counselling information on: benefits, supplementation duration, side-effects and management of side-effects were tabulated against the level of IFAS knowledge. For each content of counselling information provided, the level of IFAS knowledge was scored and categorized as high and low, both adding up to the total 100% of the respondents who were counselled on each specific content of counselling information.

The proportion of pregnant women provided with counselling information on various aspects of IFAS is shown in Figure 2. While most (80%) of the women were informed of the benefits of IFAS, half (50%) were informed on supplementation duration, a third (32%) on side-effects and only 16% on management of side-effects. The content of IFAS counselling was associated with the level of maternal IFAS knowledge. Among the respondents who received information on the benefits of IFAS, only 58.2% scored high for IFAS knowledge. However, those counselled on the side effects and the duration of IFAS supplementation demonstrated high knowledge scores of 83.1% and 77.5%, respectively. In addition, respondents who received information on the management of IFAS side effects demonstrated the highest (95%) knowledge score ( Figure 3).

Further analysis with multivariate logistic regression revealed that counselling on the duration of IFAS supplementation, its side effects and their management were the predictors of maternal IFAS knowledge. Respondents counselled on the duration of IFAS supplementation were 6.3 times more likely (AOR=6.27; 95%CI, 3.24 – 12.16; P<0.001) to have high knowledge scores. Furthermore, counselling information on IFAS that included side effects was more likely (AOR=4.5; 95%CI, 2.01 – 10.07; P<0.001) to contribute to high knowledge scores among the respondents. The respondents who were counselled on management of IFAS side-effects were 10.3 times more likely (AOR=10.31; 95%CI, 2.10 – 50.59, P=0.004) to have high knowledge scores compared to those who were not counselled. However, respondents who were counselled on the benefits of IFAS, did not show any statistical (AOR=1.48; 95%CI, 0.63 – 3.51, P=0.368) difference on the knowledge score compared to those not counselled ( Figure 3).

## Discussion

The objectives of this study were to assess maternal knowledge on iron and folic acid supplementation, availability of IFAS counselling documents in health facilities, practices and content of IFAS counselling information provided to pregnant women in health facilities and determine the association between content of counselling information and level of maternal knowledge on IFAS. The study was based at the antenatal clinic where IFAS services are provided. Health care providers, specifically nurses, working in those antenatal clinics are expected to provide IFAS counselling information together with the supplements as part of Focused Antenatal Care (FANC) to all pregnant women attending antenatal care in all public health facilities in Kenya. The Ministry of Health, through the Division of Nutrition, where micronutrient deficiency control and supplementation programmes fall, is supposed to facilitate quality counselling on IFAS by providing the appropriate job aids including IEC materials and counselling guides on IFAS.

The findings of the study showed: (i) less than 50% of the pregnant women had high IFAS knowledge level; (ii) apart from IFAS posters, other counselling guides were scarcely available in the health facilities; (iii) limited IFAS counselling information was provided to pregnant women who attended antenatal care even though they were all provided with IFAS supplements in all health facilities (iv) the content of counselling varied in different health facilities and was associated with the level of IFAS knowledge. This underscores the need to strengthen implementation of counselling for the pregnant women regarding nutrition and its importance on pregnancy outcome. Health care providers (HCPs) should be supported by the health systems to escalate counselling of pregnant women every time they meet them.

The findings reveal that less than half of the pregnant women had high IFAS knowledge level despite about two-thirds (67.3%) having heard of IFAS. This is probably associated with the lack of individualized message sharing and counselling of pregnant women on IFAS by the HCPs. In addition, emphasis on the critical role IFAS plays in pregnancy may be lacking, including support for the women with tools such as brochures. This reveals that many pregnant women do not have the details about IFAS. This could also mean that we have a huge number of women who are either ignorant of IFAS or who do not know about the supplements given to pregnant women. This is evidenced by observation performed during the counselling session where the nurse informed the mother the purpose of the supplement but not its name. Similar findings have been reported with folic acid knowledge studies in Pakistan (43%) ^46^ and the United Arab Emirates where, even though 79.1% had heard of folic acid, 46.6% had accurate knowledge on role of folic acid ^47^. This calls for more appropriate strategies to give mothers detailed IFAS information of why, when and how, beginning with actual names, importance, supplementation duration, maintaining supplementation, side-effects/challenges, and more importantly how to handle these side-effects/challenges, to increase maternal knowledge on IFAS and its compliance. Generally, high IFAS knowledge has been associated with better IFAS compliance ^20^. Hence, the need to constantly seek to improve counselling approaches so as to improve maternal knowledge on IFAS.

Scarcity of IFAS counselling documents was observed in health facilities. Though IFAS counselling was provided in all facilities and varied IFAS posters displayed at different service points, the national policy guidelines were available in only two out of the five health facilities, while health workers’ counselling guides, IFAS calendars and brochures/leaflets were not available in any of the health facilities. This paucity of IFAS counselling documents in health facilities may have led to non-standardized counselling between health facilities as well as incomplete and ineffective counselling for pregnant women. This is evidenced by the findings of the counselling session observed where it was only in one health facility that the nurse provided counselling information on IFAS side-effects and their management, and evaluated comprehension of counselling content offered to the client. Research indicates that health education plays a key role in determining uptake of health interventions ^48, 49^. However, their effectiveness is hampered by lack of relevant guides and job aids. Health workers need to be provided with requisite skills, especially counselling and interpersonal skills, and job aids to provide effective health education to clients ^50^. These include policy and counselling guides to ensure standardized health messages ^48^. As part of health education, materials for clients to take home should also be provided for their references. These guides should be regularly updated and availed on time for them to be useful. Subsequently, ensuring HCPs have the right tools on what to communicate to clients will enhance effective communication which is one of the key counselling skills essential for behaviour change including knowledge and practice.

Limited IFAS counselling information was provided to the pregnant women who attended antenatal care but they were all provided with IFAS supplements in all the health facilities. The content of counselling varied in different health facilities. Despite the universal provision of IFAS to all antenatal women, they were not counselled on the causes, features or consequences of anaemia. Moreover, counselling information on; the enhancers/inhibitors of iron/folate absorption, and the fact that there is increased nutritional requirement during pregnancy, was not provided in any of the health facilities. This is consistent with research reports that a great percentage of maternal anaemia occurs due to insufficient intake of bioavailable dietary iron particularly in developing countries ^51, 52^. It is therefore very important to ensure proper choices of food that promote iron and folate absorption among pregnant women by providing effective counselling and information on the food interactions and practices. This non-standardized counselling in health facilities resulting from lack of counselling guides may in turn have contributed to low level of IFAS knowledge and compliance as found in studies among respondents whose primary source of information was health care providers ^21, 30, 53, 54^. Accompanying communication with supplementation greatly improved compliance and decreased anaemia prevalence among adolescents in Tanzania ^26^. Research findings have reported limited knowledge/lack of information among health care providers in many aspects of education and counselling in Kenya as a set-back that adversely affects IFAS utilization ^9^ and needs to be addressed. This calls for standardization of IFAS counselling messages provided to pregnant women.

The content of counselling differed in the health facilities as discussed above and was associated with the level of IFAS knowledge. As far as content of IFAS counselling in relation to maternal knowledge is concerned, except those informed on benefits, all the other counselling aspects showed a very high difference among those who had low and high IFAS knowledge. Research indicates that experiencing side-effects is one of the reasons for low IFAS compliance ^20^. As most women stop taking iron-folate tablets due to side effects, it is important that pregnant women are effectively counselled and provided with accurate, detailed information on the side-effects and how to effectively manage them to ensure adherence to IFAS ^6, 20, 50^. Lack of counselling on the side-effects could be due to either lack of information by HCPs or their wrong attitude. While some HCPs may imagine that by mentioning side-effects of IFAS, pregnant women will not take the supplements, on the contrary, detailed counselling on the same yielded higher levels of knowledge. If women are counselled on the expected side-effects and their management and reassured they will subside with time, they are less likely to react negatively to side-effects. This was evidenced in an Indonesia study where women were not bothered by side-effects because they had been warned of their likely occurrence ^55^. This contrasts many studies that have shown side-effects as a major hindrance to IFAS compliance. Realistically, it seems like the actual problem is the quality of counselling as indicated in other studies ^50, 56^. Thus, it is important that HCPs should be well informed to enable them provide women with relevant information and conduct effective, quality counselling on IFAS to ensure compliance ^20^. This can be implemented by formally involving community health workers more in the IFAS programme for closer follow-up and counselling of the pregnant women at community level, as demonstrated in other studies ^55^.

This study had several limitations. The assessment of counselling and knowledge levels was done at one point in time by asking pregnant women whether they were counselled or not and the content of the counselling. Only one counselling session was observed in each health facility. This is a limitation of cross-sectional designs. In addition, this study relied heavily on verbal reports on IFAS, which may have introduced some recall bias and subjectivity. However, this challenge was circumvented through proper training of interviewers and double questioning that enabled identification of any inconsistencies in the reports. The study involved pregnant women who are under high hormonal influence leading to mood changes as well as pregnancy associated fatigue which may affect response and consistency. This was mitigated through message sharing, clarification, reassurance and psychosocial support of the respondents. Furthermore, participation in the study was purely voluntary and respondents were asked whether they were comfortable answering the questions and they were at liberty to discontinue. This ensured that their rights were respected and was in compliance with the ethical requirement of non-coercion. Finally, although important associations between counselling and knowledge levels are demonstrated in this study, this was a cross-sectional descriptive study whose results cannot be used to make causal inferences. Also, generalizations of the study findings to other areas may be difficult since the study involved one sub-county.

## Conclusion

Findings from this study indicate that less than half of the pregnant women had high IFAS knowledge, IFAS documents were scarce in health facilities, there was limited and varied IFAS counselling information in different health facilities and content of counselling was associated with levels of knowledge on IFAS among pregnant women. Counselling information on the duration of IFAS supplementation, IFAS side effects and their management were significantly associated with high levels of knowledge on IFAS among pregnant women. This underscores the need to strengthen focused and targeted counselling for women attending antenatal clinic to improve compliance and pregnancy outcomes. The researchers recommend provision of IFAS documents and IEC materials to peripheral health facilities, standardization of counselling content provided on IFAS and formal involvement of community health workers in the IFAS programme for closer follow-up and counselling of the pregnant women at community level. Further, intervention studies are recommended that allow for causal inferences.

## Consent

Verbal and written informed consent was provided by all the study participants before recruitment into the study.

## Data availability

The data underlying this study is available from Open Science Framework Dataset 1: Knowledge and counselling, Dataset 2: Observation checklist-counselling. DOI https://doi.org/10.17605/osf.io/x8tj3 ^33^. This dataset is available under a CCO 1.0 Universal license

**Dataset 1. Counselling information and knowledge on iron and folic acid supplementation (IFAS)**

This is the data set used for analysis using STATA version 13. It contains the information on socio-demographic information of study respondents, IFAS knowledge and counselling information, as per the attached questionnaire ( Supplementary File 1).

**Dataset 2. Observation checklist: counselling practices and content of information**

This data set contains a table showing the counselling practices and content of counselling information, as observed during an antenatal counselling session, as well as available iron and folic acid supplementation (IFAS) counselling documents in each health facility.

## Supplementary material

Supplementary File 1: Study questionnaire.

Supplementary File 2: Completed STROBE reporting checklist.

Supplementary File 3: Author responses and the changes made in this version following the reviewers’ comments.
