# Supplementary material for: Counselling and knowledge on iron and folic acid supplementation (IFAS) among pregnant women in Kiambu County, Kenya: a cross-sectional study
Source: AAS Open Res. 2019 May 13;1:21. Originally published 2018 Jul 19. [Version 3] doi: 10.12688/aasopenres.12891.3 (PMC7118767; doi:10.12688/aasopenres.12891.3)
Supplement: Supplementary file 1 [file aasopenres-1-14045-s0000.tgz › 203cad2a-db76-4fc7-9468-d6ec35b344e7.docx]

**Article title:** **Counselling on Iron and Folic Acid Supplementation (IFAS) is associated with improved knowledge among pregnant women in a rural County of Kenya**

**E-mail address of the corresponding author:** kwanjira@uonbi.ac.ke

**APPENDIX I: QUESTIONNAIRE**

**STUDY TITLE:** Iron and Folic Acid Supplementation among Pregnant Women: A Community Based Approach in Kiambu County, Kenya

Questionnaire Number _________________ Date of interview________________________

**SOCIO-DEMOGRAPHIC DATA**

1. Mother’s Age ______________ D.O.B: (DD/MM/YR) _____________________
2. Residence (Village) ___________________
3. Occupation

0 = Unemployed

1 = Housewife

2 = Casual labourer

3 = Self-employed

4 = Formal employment

5 = Others_______________

1. Highest education level

0 = No education

1= Primary

2 = Secondary incomplete

3 = Tertiary

1. Marital status

1 = Married

2 = Single

3 = Widow/Separated/Divorced

1. Religion

1 = Protestant Christian

2 = Catholic Christian

3 = Muslim

4 = Others (Specify) _________________________

1. Average income per month in Kenya shillings ________________________

1 = <10,000

2 = 10,000-30,000

3 = 30,000-50,000

4 = 50,000-100,000

5 = Above 100,000

1. Parity (Number of pregnancies) ________________________
   - 1. Number of living children ________________
     2. Number of bereaved children ____________
     3. Total children____________ **(Add the above)**
2. When was your last menstrual period (LMP) ___________________**(If known, go to Qn 12)**
3. Gestation in weeks ( as per ANC card) ____________ or Scan

**MOTHER’S KNOWLEDGE AND COUNSELLING ON IRON AND FOLIC ACID SUPPLEMENTATION (IFAS)**

1. Have you heard about IFAS? / J*e umesikia kuhusu nyongeza (tembe) za IFAS?* ________
   1. = No / *la* **(If no, skip to question 24)**
   2. = Yes / *ndio* **(If yes, answer all the questions in this section)**
2. If yes, how did you get this information? ***(*Wait for answer before probing and check all mentioned*) (tick all that apply)***

*Kama ndio, ulipata hii habari wapi?*

Yes No Health care provider *mhudumu wa afya hospitalini*

Yes No Community health worker / *mhudumu wa afya wa jamii (kijijini)*

Yes No Posters

Yes No Television / *televisheni*

Yes No Radio / *redio*

Yes No Other mothers, relatives, friends or neighbours / *Kina mama wengine, watu wa familia, rafiki au jirani*

Yes No Community leaders / *viongozi wa jiji*

Yes No Newspapers, magazines or books / *gazeti, jarida au vitabu*

Others (specify) / *zingine*______________________________

1. Have you ever received any leaflet/brochure from the health facility on IFAS? / *Je, umewahi kupata kijikaratasi chochote kuhusiana na IFAS kutoka hospitalini?*
   1. = No / *la*
   2. = Yes / *ndio*
2. Were you informed the benefits of IFAS at the health facility? / *Je, ulielezewa umuhimu wa IFAS hospitalini?*
   1. = No / *la*
   2. = Yes / *ndio*
3. What are the benefits of IFAS? /*Nielezee ni yapi manufaa ya IFAS?* ***(*Do not prompt, check all mentioned*)***
   1. = Don’t know / *sijui*

Yes No Prevents anemia among pregnant women */ Huzuia upungufu wa damu kwa kina mama wajawazito*

Yes No Protects mother from sicknesses / *hukinga mama kutokana na maradhi*

Yes No Gives mother strength during delivery / *hupea mama nguvu wakati wa kujifungua*

Yes No Increases amount of blood / *huongeza kiwango cha damu*

Yes No Makes foetus grow healthy and strong / *hupea mototo aliye ndani ya tumbo la mama nguvu na afya*

Yes No Others (Specify) / *zingine*______________________

1. How often should IFAS be taken? / *Nielezee jinsi ya kutumia kutumia IFAS?*
   1. = Do not know /*sijui*
   2. = Once every week / *mara moja* *kwa wiki*
   3. = Once daily / *mara moja kila siku ****
   4. = 2-4 times a day / *mara mbili hadi nne kwa siku*
   5. = Others (specify) / *zingine*____________________
2. Were you informed for how long you should take IFAS at the health facility? *Je, ulielezewa unastahili kutumia IFAS kwa mda gani hospitalini?*
   1. = No / *la*
   2. = Yes / *Ndio*
3. For how long should you take IFAS? / *Nielezee unastahili kutumia IFAS kwa mda gani?*
   1. = Don’t know / *sijui*
   2. = Before becoming pregnant */ kabla ya kupata mimba*
   3. = At least 3 months */ kwa miezi kama mitatu hivi*
   4. = Throughout pregnancy */ wakati wote wa uja uzito ****
   5. = During pregnancy and 6 weeks after delivery /*wakati wa uja uzito hadi wiki sita baada ya kujifungua*
   6. = Others (Specify) / *zingine* _______________
4. Have you been informed that you can experience some side-effects from taking IFAS? / *Ulielezewa ya kwamba unaweza kupata madhara baada ya kutumia IFAS*
   1. = No / *la*
   2. = Yes / *ndio*
5. What are the side-effects of IFAS */ Ni yapi madhara ya kutumia IFAS?* (**Check all mentioned*)***

0 = None /Don’t know / *sijui*

Yes No Epigastric pain */ maumivu ya tumbo*

Yes No Abdominal pain / *kuumwa* na tumbo

Yes No Nausea */ kichefuchefu*

Yes No Vomiting / *kutapika*

Yes No Diarrhoea */ kuendesha*

Yes No Constipation */ kuvimbiwa*

Yes No Faeces may turn black */ kinyesi kugeuka rangi na kuwa cheusi*

Others (specify) / *zingine*____________________

1. Were you informed how you can manage the side-effects at the health facility */ Je, ulielezewa unawezaje kukabiliana na madhara haya hosipitalini?*
   1. = No / *la*
   2. = Yes / *ndio*
2. How can you manage the side-effects */ Nielezee unawezaje kukabiliana na madhara haya?* (**Check all mentioned*)***

0 = Don’t know / *sijui*

Yes No Avoid taking high dose Vitamin C supplements together with IFA tablet */ epuka kutumia kiasi kikubwa cha Vitamini C supplement, pamoja na vidonge vya IFAS*

Yes No Eat plenty of fruits and vegetables */ kula matunda na mboga kwa wingi*

Yes No Take IFAS with meals */ meza IFAS wakati wa kula chakula*

Yes No Take IFAS while going to bed */ meza IFAS unapoenda kulala*

Yes No Stop using IFAS / *acha kutumia IFAS*

Yes No Go back to hospital / *rudi hospitali*

Others (specify) / *zingine*____________________

1. What happens if you do not get enough iron and folic acid in the body during pregnancy? **(Prompt and** **Check all mentioned*)***

0 = No effect / *hakuna*

Yes No Anaemia/ Low blood levels / *upungufu wa damu*

Yes No Excessive bleeding during pregnancy/delivery */ kuvuja damu zaidi unapojifungua*

Yes No Baby gets congenital malformations / *mtoto hupata shida za kimaumbile*

Yes No Baby gets mental disability */ mtoto hupata upungufu wa akili*

Yes No Low birth weight baby */ kilo za mtoto za kuzaliwa hupungua*

Yes No Preterm baby / *mtoto huzaliwa kabla ya umri wake kufika*

Others / *zingine* ____________________

1. What are the signs and symptoms of anaemia (low blood levels) */ Nielezee dalili za upungufu wa damu mwilini ni gani?* (**Check all mentioned*)***

0 = Don’t know / *sijui*

Yes No Feels weak */ udhaifu*

Yes No Looks pale */ kuchujuka rangi kwa kope za macho na viganja*

Yes No Palpitations */ kutoweza kupumua vyema*

Yes No Headaches */ kuumwa na kichwa*

Yes No Dizziness / *kisunzi*

Yes No Tiredness and easily fatigued /*uchovu*

Yes No Swells legs /*uvimbe kwa miguu*

Others (specify) / *zingine* ________________

1. Can you tell me some food sources that increase the blood levels in the body during pregnancy? (**Check all mentioned*)***

*Niambie vyakula vyenye iron kwa wingi*________

0 = Don’t know / *sijui*

Yes No Liver / *ini*

Yes No Red meat e.g. beef / *nyama nyekundu* *kama ya ng’ombe*

Yes No White meat e.g. chicken, fish / *nyama nyeupe kama kuku,* *samaki*

Yes No Dark-green leafy vegetables / *mboga zenye majani ya rangi ya kijani kibichi*

Yes No Whole grain cereals e.g. maize, sorghum */ mbegu nzima kama mahindi, mtama*

Yes No Legumes e.g. beans, peas */ mbegu kama maharagwe, njahi, minji*

Yes No Eggs */ mayai*

Others (Specify) / *zingine*________________________
