# Supplementary material for: Counselling and knowledge on iron and folic acid supplementation (IFAS) among pregnant women in Kiambu County, Kenya: a cross-sectional study
Source: AAS Open Res. 2019 May 13;1:21. Originally published 2018 Jul 19. [Version 3] doi: 10.12688/aasopenres.12891.3 (PMC7118767; doi:10.12688/aasopenres.12891.3)
Supplement: Supplementary file 3 [file aasopenres-1-14045-s0002.tgz › 2d9822b8-9dfe-4943-b626-0e49c727136c_Supplementary_file_3.docx]

**CHANGES MADE TO MANUSCRIPT FOLLOWING REVIEWER’S COMMENTS**

The table below gives a detailed response of each of the reviewer’s comments.

**A TABLE OF CHANGES AGAINST EACH POINT RAISED BY REVIEWERS**

| **REVIEWER’S COMMENTS** | **RESPONSE** |
| --- | --- |
| **REVIEWER 1 - Jacqueline K. Kung'u** |  |
| 1. **Introduction:**   Include a paragraph in the introduction on the role of behaviour change and communication strategies including counselling on health outcomes including maternal knowledge.  1. Importance of contextually tailored strategies - what strategies exist in Kenya? Are they evidence/research based and are they integrated within programs. 2. What are the clear BCC/Counselling goals and objectives and are they achievable within programs - find studies that have reported this.  3. What affects delivery of BCC/Counselling e.g. availability of IEC material, training, motivation, workload of the health worker etc - find studies that have reported this | The introduction has been reviewed to include information on the role of behaviour change and communication strategies including counselling on health outcomes including maternal knowledge, as outlined by the reviewer. |
| 1. **Methods:**    1. Provide a brief description of what a functional community unit means and implications to this study | A functional community unit is the criteria that was used to select the Sub-County for the study since not all Sub-counties could be involved. A functional community unit means its community health volunteers are active in health care provision activities. This implies is completeness in health care provision at the community level which is the first level of health care service delivery thus there is no gap in iron and folic acid supplementation services at the community level where the pregnant women reside. |
| - 1. Was the tool used generic tool or was it developed specifically for this study. If the latter then a brief description is required to qualify it and for replication of other studies. What were the considerations in developing it, do we know its reliability etc.   2. If the tool used was developed for this study I want to suggest that there is a lot missing to qualify the methods – the authors need to include a factor analysis based on principal component extraction followed by varimax rotation to examine the structure of the items in the scale used. Internal consistency of the scale should be investigated through Cronbach’s alpha coefficient. If the questionnaire is reliable, no item should cause a substantial decrease in the alpha value. Cronbach’s alpha reliability coefficient ranges between 0 and 1; the closer the Cronbach’s coefficient is to 1, the greater the internal consistency of the items in the scale. A coefficient ≥0.7 is considered acceptable. Please consult a statistician for this analysis - it will make the paper stronger. Otherwise as is there are glaring gaps in the analysis | The tool used was developed for this study. It was pre-tested at Kiambu level 5 hospital.  To ensure reliability of the questionnaire, a test re-test method was adopted in pre-testing, whereby a repeat pre-test was conducted after two weeks, and Cohen’s kappa statistic was used to measure the level of agreement of the results from the two pre-tests. All the questions repeated had a kappa value of above 0.7 after comparison thus the questionnaire was considered reliable, hence all the questions were retained.  To ensure validity of the questionnaire, it was shared and discussed with experts from the Ministry of Health, division of nutrition, and the study supervisors. The feedback obtained from these experts and pre-testing results was used to refine the tool and improve its quality to ensure the questions were able to test what was intended.  The questionnaire used in the study is attached in supplementary file 1 and does not contain a likert scale. |
| 1. **Discussion**    1. Begin by describing the ideal situation in a Kenyan health facility in terms of IEC and BCC material for IFAS and what the health worker is required to do during the encounter with the mother. This then sets the stage for your findings.    2. Make sure that everything being discussed is a finding from your study and there needs to be a clear distinction between a study finding and an inference being made.    3. Some incorrect statements e,g, mother may not know the actual name but may relate with a name in  language that the mother is familiar with  or  some studies show that the women may not know the name but if you describe IFAS as the medicine that adds blood during pregnancy - a lot more women can identify. Therefore it’s very important how the question was asked. | The discussion has been revised to describe the ideal situation in a Kenyan health facility in terms of IEC and BCC materials for IFAS and what the health worker is required to do during the encounter with the mother to set the stage for the findings.  The study findings have been discussed appropriately and inferences clearly distinguished.  Confusing statements have been corrected |
|  |  |
| **REVIEWER 2 – Anselimo Makokha** |  |
| 1. The title should mention the specific county. | The specific County (Kiambu) has been mentioned in the title |
| 1. **Methods:** 2. Since the study is essentially testing the association between counselling of the mothers and knowledge level of IFAS, the design should have included the experimental group who received the counselling and a control group who did not receive the counselling. Otherwise it becomes difficult to determine if any changes in the knowledge status were not due to chance.   Despite this observations the paper may still be suitable for indexing if it focuses simply on a cross sectional study of the knowledge level of IFAS for women who had received counselling, without attempting to establish the effect of counselling on knowledge level of IFAS since there was no control group   1. The methods should also include what the minimum sample size was and its basis. 2. Further how the counselling was done is not comprehensively described, including for what period and how frequently. 3. The sub county and the five public health facilities where the study was carried out should be identified. | The study design was a cross-sectional and there was no control group, so the study did not attempt to establish the effect of counselling on knowledge level of IFAS.  This has been corrected right from the title which has been adjusted to read as “Counselling and knowledge on iron and folic acid supplementation (IFAS) among pregnant women in Kiambu County”  The minimum sample size was 285, as determined using Fisher’s formula (1999) [n = Z2pq/e2] for a cross-sectional study, using the prevalence of IFAS compliance obtained from Thika hospital, Kiambu County among women attending ANC clinic, of 24.5%.  This being a cross-sectional study, there was no intervention done for a certain duration on counselling, rather the status of whether counselling on IFAS was offered or not during ANC was determined and the content of counselling offered determined as per specific objective 2 and questionnaire attached in supplementary file 1.  The Sub-County and five facilities where the study was carried out have been indicated as: Lari Sub-County and Lari, Githirioini, Kagwe, Kagaa and Kinale health facilities |
| 1. **Results**   In the Results (Par.2), if the cut off for high or low knowledge level was the median of the scores, then there should have been an equal number of those who scored high and those who scored level knowledge level. However, it is reported that only 40.9% scored high level. This needs explanation. Irrespective of the explanation, choice of the median as the cut-off point to classify knowledge level may be inappropriate as it will simply divide the group into two equal parts, and may not reflect the true status of the level of knowledge on IFAS. | The median was used in this study because on computation of the scores, the mean was slightly different from the median, meaning that the distribution of scores was not exactly normal. A further test of normality was done to determine the distribution of maternal knowledge scores using Shapiro-Wilk W test which showed that the distribution of maternal knowledge scores was not normal hence the median was used as the cut-off between high and low IFAS knowledge (Sadore et al, 2015) |
| 1. **Conclusion**   Without a control group the conclusion that counselling information on certain aspects of IFAS was a predictor of knowledge on IFAS may not be valid. | The statement has been corrected to reflect what is achievable in a cross sectional study |
| 1. There are also some grammatical mistakes in the manuscript | The manuscript has been proof-read and grammatical mistakes corrected |
|  |  |
| **REVIEWER 3 - Jacob K. Kariuki** |  |
| 1. **Methods:** 2. In the methods section the authors are already outlining the predictors and effect modifier of maternal IFAS knowledge. These data belong to the results section since you could not have known this apriori. The methods section should focus on discussing how the study and analyses were done. | The information on predictors and effect modifier of maternal knowledge has been removed from methods section and taken to results section as advised. |
| 1. Using your sample median as the cut off for IFAS knowledge is a major flaw in the analysis. Consider redoing your analyses using a literature based cut off. You can do this by referencing a well-designed study on the topic. | The median was used in this study because on computation of the scores, the mean was slightly different from the median, meaning that the distribution of scores was not exactly normal. A further test of normality was done to determine the distribution of maternal knowledge scores using Shapiro-Wilk W test which showed that the distribution of maternal knowledge scores was not normal hence the median was used as the cut-off between high and low IFAS knowledge (Sadore et al, 2015) |
| 1. The methodology section does a good job describing how the regression analyses were done. It would have been also helpful to see a description of the protocols that were used to determine availability of IFAS counselling documents in health facilities since this was the first objective of the study. The checklist you have provided in the supplemental material simply solicits the opinion of the study participants about the availability of the resources. Under dataset 2, you allude to an observation by the researcher during antenatal visit. While this observation would help verify of the counselling documents are used, it may not determine if the documents are available in the facility. It is very important to be clear if you did any objective audit. | I wish to clarify that an objective audit was actually done.  Using the observation checklist, the researcher actually physically observed which IFAS documents were available at the health facility and ticked those available on the checklist. With the help of the facility in charge, the researcher determined if there were any other documents not in the vicinity and further requested to see all the documents each facility had on IFAS. The researcher then went ahead to observe a counselling session at each facility and used the checklist to tick what she observed. |
| 1. **Results** 2. Under results, it would be helpful to see if awareness varied by parity and prior antenatal visits. Since participants above 40 yrs reported more awareness, one is tempted to ask why? The statistics you have in Table 1 are not helpful because they reference the sample median. After addressing the issues raised under methodology, analysis that includes parity could help reveal any confounding due to previous antenatal visits. It also helps to test the significance of the differences. | The multiple regression results relating maternal IFAS knowledge with the socio-demographic factors have been published in another article from this study (Kamau et al, 2019) |
| 1. Under results, you present the results of observed counselling practices, but there is no information about how many observations were done and how they were distributed across facilities since you employed cluster sampling. Figure 1 is not very informative. | This being a cross-sectional study, one counselling session was observed in each health facility, as indicated in methods section |
| 1. It is appropriate to examine the relationship between knowledge and other factors included in the regression, but it is important to emphasize that the results do not infer causation.  Consider adding this to the limitations of the study | The emphasis that the results on the relationship between knowledge and counselling do not infer causation has been added to limitations |
| 1. **Discussion**   Consider reframing your discussion around the 3 aims of the study. That will make it more coherent with the rest of the study | The discussion has been revised around the aims of the study to make it more coherent with the rest of the study. |
