# Supplementary material for: Counselling and knowledge on iron and folic acid supplementation (IFAS) among pregnant women in Kiambu County, Kenya: a cross-sectional study
Source: AAS Open Res. 2019 May 13;1:21. Originally published 2018 Jul 19. [Version 3] doi: 10.12688/aasopenres.12891.3 (PMC7118767; doi:10.12688/aasopenres.12891.3)
Supplement: Peer review history

# Peer review: Reviewer response for version 3

**DOI:** 10.21956/aasopenres.14045.r26941

**Published:** 2019-05-14

**Authors:** Anselimo Makokha

**Affiliations:** 1 Department of Food Science and Technology, Jomo Kenyatta University of Agriculture and Technology (JKUAT), Nairobi, Kenya

The authors have adequately addressed the concerns that had been raised. The article is approved for indexing.

I confirm that I have read this submission and believe that I have an appropriate level of expertise to confirm that it is of an acceptable scientific standard.

# Peer review: Reviewer response for version 2

**DOI:** 10.21956/aasopenres.14035.r26841

**Published:** 2019-04-10

**Authors:** Jacqueline K Kung'u

**Affiliations:** 1 Nutrition International, Nairobi, Kenya

I have no additional comments for this manuscript. The authors have addressed my concerns and the manuscript can be indexed as is.

I confirm that I have read this submission and believe that I have an appropriate level of expertise to confirm that it is of an acceptable scientific standard.

# Peer review: Reviewer response for version 2

**DOI:** 10.21956/aasopenres.14035.r26839

**Published:** 2019-04-02

**Authors:** Anselimo Makokha

**Affiliations:** 1 Department of Food Science and Technology, Jomo Kenyatta University of Agriculture and Technology (JKUAT), Nairobi, Kenya

1. In the Introduction the first objective should be to "Determine maternal knowledge on iron and folic acid supplementation (IFAS) among pregnant women who had received counselling".
  As in my earlier comments it may not be possible to address the 4th objective of determining the association between content of counselling information and level of maternal knowledge on IFAS as there was no control group. The other three objectives should suffice.
2. In the Methods, the square part appears as 2?
3. The cross tabulation and determination of association should be between knowledge and sociodemographic characteristics, but not between knowledge versus content of counselling information, since there was no control group that did not receive counselling.
4. In the Results, the first paragraph, specifying the variables, should be in the Methods.
5. In the second paragraph of the Results, there is a reference to the null hypothesis that "distribution of maternal knowledge scores was normal…". Was there such a null hypothesis in the first place?
  As in my first report, the issue of the use of median in determining the knowledge level, shouldn't those with high IFAS knowledge be equal to those with low IFAS knowledge, since the median is the middle entry from the lowest to the highest? It is still not clear how then those with high are 40.9%, and not 50%.
6. In the Results, the authors should omit the section on the relationship between IFAS counselling content and maternal knowledge as explained in my comments on the Introduction above.
7. Conclusion: First sentence "...pregnant women had high IFAS...". Shouldn't it be IFAS knowledge?
8. Again the authors should omit the association of counselling information and consequences with knowledge level, as there was no control group.

I confirm that I have read this submission and believe that I have an appropriate level of expertise to confirm that it is of an acceptable scientific standard, however I have significant reservations, as outlined above.

## References

[1] : Referee Report For: Counselling and knowledge on iron and folic acid supplementation (IFAS) among pregnant women in Kiambu County, Kenya: a cross-sectional study [version 1]. *AAS Open Research*.1(21) : Reference source 10.12688/aasopenres.12891.2PMC711876732259021

# Response

**Published:** 2019-04-20

**Authors:** Mary Kamau

**Affiliations:** University of Nairobi, Kenya

Thank you very much Professor for your review and comments. We have addressed them accordingly.

We wish to clarify that this was a cross-sectional study, looking at the counselling status and knowledge on iron and folic acid supplementation (IFAS) among pregnant women in Kiambu County. We agree that there was no control group. We wish to clarify that it was not an intervention and we were not providing counselling services in the study but were simply looking at the counselling status as it were in the health facilities: whereby some of the pregnant women had been counselled on various components of IFAS whereas others had not been counselled at all. So there was no control group since it was a one-time observation study but some of the pregnant women did not receive any counselling. The fact that some had been counselled while others had not, formed the basis for all our comparisons and associations in this study.

As earlier explained, the median was used in this study as the cut-off between high and low IFAS knowledge (Sadore *et al.*, 2015) because on computation of the scores, the mean was slightly different from the median, meaning that the distribution of scores was not exactly normal. A further test of normality was done to determine the distribution of maternal knowledge scores using Shapiro-Wilk W test which showed that the distribution of maternal knowledge scores was not normal hence the median was used as the cut-off between high and low IFAS knowledge (Sadore *et al.*, 2015).

The null hypothesis referred to here is not about this study but about the statistical test of normality Shapiro-Wilk W test. Sorry if this confused you. Therefore, those with high IFAS knowledge cannot be equal to those with low IFAS knowledge in this case because the median and the mean are not the same. References where the median has been used as the cut-off in other studies is given as well as further statistical tests done to prove this, as stated above.

Hope we have clarified it all.

Once again, thank you very much!

# Peer review: Reviewer response for version 1

**DOI:** 10.21956/aasopenres.13959.r26561

**Published:** 2018-12-18

**Authors:** Jacob K. Kariuki

**Affiliations:** 1 School of Nursing, University of Pittsburgh, Pittsburgh, PA, USA

The stated aims of the study are timely and very appropriate. Below are some of the issues that need to be addressed:

 1. In the methods section the authors are already outlining the predictors and effect modifier of maternal IFAS knowledge. These data belong to the results section since you could not have known this apriori. The methods section should focus on discussing how the study and analyses were done.

 2. Using your sample median as the cut off for IFAS knowledge is a major flaw in the analysis. Consider redoing your analyses using a literature based cut off. You can do this by referencing a well designed study on the topic.

 3. The methodology section does a good job describing how the regression analyses were done. It would have been also helpful to see a description of the protocols that were used to determine availability of IFAS counselling documents in health facilities since this was the first objective of the study. The checklist you have provided in the supplemental material simply solicits the opinion of the study participants about the availability of the resources. Under dataset 2, you allude to an observation by the researcher during antenatal visit. While this observation would help verify of the counselling documents are used, it may not determine if the documents are available in the facility. It is very important to be clear if you did any objective audit.

 4. Under results, it would be helpful to see if awareness varied by parity and prior antenatal visits. Since participants above 40 yrs reported more awareness, one is tempted to ask why? The statistics you have in Table 1 are not helpful because they reference the sample median. After addressing the issues raised under methodology, analysis that includes parity could help reveal any confounding due to previous antenatal visits. It also helps to test the significance of the differences.

 5. Under results, you present the results of observed counselling practices, but there is no information about how many observations were done and how they were distributed across facilities since you employed cluster sampling. Figure 1 is not very informative.

 6. It is appropriate to examine the relationship between knowledge and other factors included in the regression, but it is important to emphasize that the results do not infer causation.  Consider adding this to the limitations of the study.

 7. Consider reframing your discussion around the 3 aims of the study. That will make it more coherent with the rest of the study.

 Overall thoughts: This paper addresses an important topic in a region that is understudied. Addressing the flaws identified above will improve its quality and contribution to science.

I confirm that I have read this submission and believe that I have an appropriate level of expertise to confirm that it is of an acceptable scientific standard, however I have significant reservations, as outlined above.

# Peer review: Reviewer response for version 1

**DOI:** 10.21956/aasopenres.13959.r26644

**Published:** 2018-12-04

**Authors:** Anselimo Makokha

**Affiliations:** 1 Department of Food Science and Technology, Jomo Kenyatta University of Agriculture and Technology (JKUAT), Nairobi, Kenya

The title should mention the specific county.

 Since the study is essentially testing the association between counseling of the mothers and knowledge level of IFAS, the design should have included the experimental group who received the counseling and a control group who did not receive the counseling. Otherwise it becomes difficult to determine if any changes in the knowledge status were not due to chance. The methods should also include what the minimum sample size was and its basis. Further how the counseling was done is not comprehensively  described, including for what period and how frequently. The sub county and the five public health facilities where the study was carried out should be identified.

 In the Results (Par.2). if the cut off for high or low knowledge level was the median of the scores, then there should have been an equal number of those who scored high and those who scored level knowledge level. However, it is reported that only 40.9% scored high level. This needs explanation. Irrespective of the explanation, choice of the median as the cut off point to classify knowledge level may be inappropriate as it will simply divide the group into two equal parts, and may not reflect the true status of knowledge level.

 Without a control group the conclusion that counseling information on certain aspects of IFAS was a predictor of knowledge on IFAS may not be valid.

 Despite this observations the paper may still be suitable for indexing if it focuses simply on a cross sectional study of the knowledge level of IFAS for women who had received counseling, without attempting to establish the effect of counseling on knowledge level of IFAS since there was no control group.

 There are also some grammatical mistakes in the manuscript.

I confirm that I have read this submission and believe that I have an appropriate level of expertise to confirm that it is of an acceptable scientific standard, however I have significant reservations, as outlined above.

# Response

**Published:** 2018-12-18

**Authors:** Mary Kamau

**Affiliations:** University of Nairobi, Kenya

Thank you very much for taking your time to review my manuscript.

I highly appreciate your comments. I will work on them.

Once again, thank you!

# Peer review: Reviewer response for version 1

**DOI:** 10.21956/aasopenres.13959.r26614

**Published:** 2018-11-02

**Authors:** Jacqueline K Kung'u

**Affiliations:** 1 Nutrition International, Nairobi, Kenya

**Introduction**

 The central theme of this study is counselling. What is definitely missing in this introduction is the role of behaviour change and communication strategies including counselling on health outcomes including maternal knowledge. The authors need to include a paragraph on this in the introduction.

 1. Importance of contextually tailored strategies - what strategies exist in Kenya? Are they evidence/research based and are they integrated within programs.

 2. What are the clear BCC/Counselling goals and objectives and are they achievable within programs - find studies that have reported this.

 3. What affects delivery of BCC/Counselling e.g. availability of IEC material, training, motivation, workload of the health worker etc - find studies that have reported this.

**Methods**

- Provide a brief description of what a functional community unit means and implications to this study.
- Was the tool used generic tool or was it developed specifically for this study. If the latter then a brief description is required to qualify it and for replication of other studies. What were the considerations in developing it, do we know its reliability etc.
- If the tool used was developed for this study I want to suggest that there is a lot missing to qualify the methods – the authors need to include a factor analysis based on principal component extraction followed by varimax rotation to examine the structure of the items in the scale used. 
- Internal consistency of the scale should be investigated through Cronbach’s alpha coefficient.  
- If the questionnaire is reliable, no item should cause a substantial decrease in the alpha value. Cronbach’s alpha reliability coefficient ranges between 0 and 1; the closer the Cronbach’s coefficient is to 1, the greater the internal consistency of the items in the scale. A coefficient ≥0.7 is considered acceptable.

Please consult a statistician for this analysis - it will make the paper stronger. Otherwise as is there are glaring gaps in the analysis. 

**Discussion**

- Begin by describing the ideal situation in a Kenyan health facility in terms of IEC and BCC material for IFAS and what the health worker is required to do during the encounter with the mother. This then sets the stage for your findings.
- Make sure that everything being discussed is a finding from your study and there needs to be a clear distinction between a study finding and an inference being made.
- Some incorrect statements e,g, mother may not know the actual name but may relate with a name in  language that the mother is familiar with  or  some studies show that the women may not know the name but if you describe IFAS as the medicine that adds blood during pregnancy - a lot more women can identify. Therefore it’s very important how the question was asked.

 

**Conclusion**

 The cross sectional design of this study cannot infer this so this statement is definitely erroneous “ *Content of counselling substantially contributed to high levels* *of knowledge on IFAS among pregnant women”. * What I would rather you say is that....There was an association between the content of counselling and high knowledge.... you cannot infer causality.

I confirm that I have read this submission and believe that I have an appropriate level of expertise to state that I do not consider it to be of an acceptable scientific standard, for reasons outlined above.
